# Distinct *FLT3* Pathways Gene Expression Profiles in Pediatric De Novo Acute Lymphoblastic and Myeloid Leukemia with *FLT3* Mutations: Implications for Targeted Therapy

**DOI:** 10.3390/ijms25179581

**Published:** 2024-09-04

**Authors:** Lizhen Zhao, Hongbo Chen, Fengli Lan, Jinjin Hao, Wenzhi Zhang, Ying Li, Yuhong Yin, Minchun Huang, Xiaoyan Wu

**Affiliations:** Department of Pediatrics, Union Hospital, Tongji Medical College, Huazhong University of Science and Technology, Wuhan 430022, China

**Keywords:** *FLT3* mutation, gene expression, clinical features, prognosis, pediatric, acute lymphoblastic leukemia, acute myeloid leukemia

## Abstract

Activating *FLT3* mutations plays a crucial role in leukemogenesis, but identifying the optimal candidates for *FLT3* inhibitor therapy remains controversial. This study aims to explore the impacts of *FLT3* mutations in pediatric acute lymphoblastic leukemia (ALL) and acute myeloid leukemia (AML) and to compare the mutation profiles between the two types to inspire the targeted application of *FLT3* inhibitors. We retrospectively analyzed 243 ALL and 62 AML cases, grouping them into *FLT3*-mutant and wild-type categories, respectively. We then assessed the associations between *FLT3* mutations and the clinical manifestations, genetic characteristics, and prognosis in ALL and AML. Additionally, we compared the distinct features of *FLT3* mutations between ALL and AML. In ALL patients, those with *FLT3* mutations predominantly exhibited hyperdiploidy (48.6% vs. 14.9%, *p* < 0.001) and higher *FLT3* expression (108.02 [85.11, 142.06] FPKM vs. 23.11 [9.16, 59.14] FPKM, *p* < 0.001), but lower expression of signaling pathway-related genes such as *HRAS*, *PIK3R3*, *BAD*, *MAP2K2*, *MAPK3*, and *STAT5A* compared to *FLT3* wild-type patients. There was no significant difference in prognosis between the two groups. In contrast, AML patients with *FLT3* mutations were primarily associated with leucocytosis (82.90 [47.05, 189.76] G/L vs. 20.36 [8.90, 55.39] G/L, *p* = 0.001), *NUP98* rearrangements (30% vs. 4.8%, *p* = 0.018), elevated *FLT3* expression (74.77 [54.31, 109.46] FPKM vs. 34.56 [20.98, 48.28] FPKM, *p* < 0.001), and upregulated signaling pathway genes including *PIK3CB*, *AKT1*, *MTOR*, *BRAF*, and *MAPK1* relative to *FLT3* wild-type, correlating with poor prognosis. Notably, internal tandem duplications were the predominant type of *FLT3* mutation in AML (66.7%) with higher inserted base counts, whereas they were almost absent in ALL (6.3%, *p* < 0.001). In summary, our study demonstrated that the forms and impacts of *FLT3* mutations in ALL differed significantly from those in AML. The gene expression profiles of *FLT3*-related pathways may provide a rationale for using *FLT3* inhibitors in AML rather than ALL when *FLT3* mutations are present.

## 1. Introduction

The Fms-like tyrosine kinase 3 (*FLT3*) gene, located on chromosome 13q12 [1], encodes a receptor crucial for the survival, proliferation, and differentiation of hematopoietic cells in both myeloid and lymphoid lineages [2]. The *FLT3* receptor comprises four regions: an extracellular region, a transmembrane portion, a juxtamembrane domain (JMD), and an intracellular C-terminal region containing a tyrosine kinase domain (TKD), which includes TKD1 and TKD2 [2]. *FLT3*-activating mutations typically involve either internal tandem duplications (*FLT3*-ITD) in the JMD, leading to ligand-independent dimerization and activation, or point mutations in the TKD (*FLT3*-TKD) resulting in constitutive receptor activation. These mutations initiate a cascade of downstream signaling pathways, including *PI3K*/*AKT*, *RAS*/*MAPK*, and *STAT5*, promoting cellular proliferation and inhibiting apoptosis [2]. Over the past decade, advancements in DNA sequencing technologies, particularly next-generation sequencing (NGS), have significantly improved the survival rates for children with leukemia. A growing number of novel leukemogenic mutations have been identified, many of which exhibit sensitivity to *FLT3* inhibitors at the cellular level [3,4,5]. However, the clinical significance of these non-canonical mutation sites in pediatric leukemia remains largely unexplored.

In acute myeloid leukemia (AML), the frequency of *FLT3*-ITD and TKD ranges from 9.7% to 16.5% and from 4.3% to 11.3%, respectively [6,7,8]. Notably, *FLT3*-ITD is linked to a deleterious prognosis, with a high *FLT3*-ITD allelic ratio indicating a particularly poor outcome in pediatric AML patients [9], and research on *FLT3*-TKD mutations shows inconsistent prognostic implications [6,10]. Meanwhile, in acute lymphoblastic leukemia (ALL), the mutation rate of *FLT3* varies from 4.7% to 6.8% [3,11]. In certain subtypes of ALL, such as high hyperdiploidy and *BCR-ABL1*-like, there is an elevated frequency of *FLT3* mutations [12,13]. Some research indicates a potential link to prognosis [14,15], while other studies on *FLT3* mutations suggest that there may not be an impact on outcome in pediatric ALL [11,16,17]. Moreover, several studies [3,17] have demonstrated that the prognosis is influenced not only by *FLT3* mutations per se but also significantly by the expression levels of *FLT3,* which are critically linked to patient outcomes [15,18,19]. Additionally, the oncogenesis of *FLT3*-ITD in AML is driven not merely by this specific mutation but also by a complex interplay of epigenetic modifications [20]. Consequently, why the identical *FLT3* activating mutations lead to markedly divergent outcomes in ALL compared to AML remains an intriguing enigma.

Sorafenib is an oral, multi-targeted tyrosine kinase inhibitor. A study demonstrated that incorporating sorafenib into conventional chemotherapy significantly enhances overall survival (OS) in pediatric patients with *FLT3*-ITD-positive AML [21]. In contrast, evidence indicated that adding sorafenib to standard chemotherapy did not produce a response in patients with *FLT3* mutations in ALL [5]. Furthermore, the data suggested that targeting *FLT3* pathways was a viable therapeutic strategy in T-cell ALL with *PRC2* mutations [22]. Consequently, despite these advancements, the mechanisms by which *FLT3* mutations drive leukemogenesis and the identification of patients who could potentially benefit from *FLT3* inhibitors remain insufficiently elucidated.

Considering the role of *FLT3* in the pathogenesis of ALL and AML and the fact that identifying the beneficiaries of *FLT3* inhibitors remains an enigma, we conducted a comprehensive analysis of pediatric ALL and AML cases. Specifically, we examined disease characteristics at initial diagnosis, co-mutations, related gene expression patterns, and clinical outcomes.

## 2. Results

### 2.1. Association of FLT3 Gene Mutations with Clinical Characteristics and Remission Status

This study included a total of 305 patients with documented genetic data: 243 with ALL and 62 with AML. Among these, 35 ALL patients (14.4%) and 20 AML patients (32.3%) harbored *FLT3* mutations.

Among the cohort of 243 ALL patients, 215 cases (88.5%) had B-cell ALL (B-ALL), of which 35 were found to have *FLT3* mutations. Interestingly, there was a notable increase in hyperdiploidy cases (48.6% vs. 14.9%, *p* < 0.001) and a decrease in *ETV6-RUNX1* fusion transcript cases (0.0% vs. 18.8%, *p* = 0.005) in the *FLT3* mutation group. Additionally, the proportion of patients achieving minimal residual disease (MRD)-negative status on day 19 was significantly lower in the *FLT3* mutation subgroup compared to the *FLT3* wild-type subgroup (21.2% vs. 45.3%, *p* = 0.009). A detailed comparison between the mutation and non-mutation subgroups is presented in Tables S1 and S2.

Among the AML patients included in the study, the presence of an *FLT3* mutation was associated with a significantly elevated white blood cell count at diagnosis (82.90 G/L vs. 20.36 G/L, *p* = 0.001). Furthermore, the *FLT3* mutation group exhibited a higher prevalence of the *NUP98* fusion transcript (30.0% vs. 4.8%, *p* = 0.018) and a lower frequency of the *AML1-ETO* fusion transcript (10.0% vs. 42.9%, *p* = 0.010). Notably, there were no significant differences observed in the treatment response. Additional details regarding these findings can be found in Tables S3 and S4.

### 2.2. The Characteristics of FLT3 Mutations

**Figure 1:**
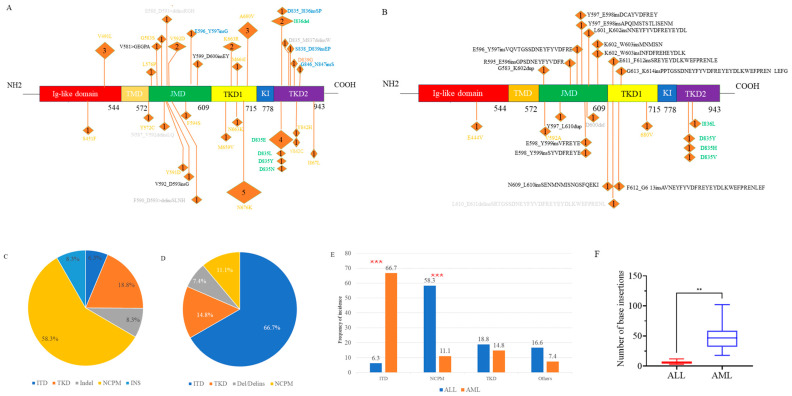
The spectrum of *FLT3* mutations in ALL (**A**) and in AML (**B**). Proportion of each mutation type in ALL (**C**) and in AML (**D**). Comparison of the proportions of each mutation type between ALL and AML (**E**). Comparison of the inserted base counts between ALL and AML (**F**). ALL, acute lymphoblastic leukemia; AML, acute myeloid leukemia. JMD, juxtamembrane domain; TMD, trans-membrane domain; TKD1, tyrosine kinase domain 1; TKD2, tyrosine kinase domain 2; KI, Kinase insert. In Figures (**A**,**B**), the colors orange, green, and black indicate non-canonical point mutations (NCPM), canonical tyrosine kinase domain (TKD) mutations, and internal tandem duplications (ITD), respectively. In Figure (**A**), gray and blue represent insertion/deletion (Indel) and insertion (INS), respectively, while in Figure (**B**), gray denotes deletion or deletion/insertion. The numbers in parentheses represent the number of patients with the mutation. ITD mutations in three AML patients were identified through transcriptome sequencing, but the specific loci are unknown and therefore are not shown in the Figure (**B**). *** *p* < 0.001. ** *p* < 0.01.

We identified a total of 48 *FLT3* mutations in 35 ALL patients, encompassing various mutation types. Among the 20 AML patients, 27 *FLT3* mutations were found (**Figure 1**A,B). Notably, the spectrum of mutations differed between ALL and AML. In ALL patients, ITD and canonical TKD mutations constituted only 6.3% and 18.8% of *FLT3* mutation events, respectively. Conversely, these mutations were predominant in AML patients, accounting for 66.7% (*p* < 0.001) and 14.8% of events, respectively (**Figure 1**C–E).

Additionally, NGS revealed numerous novel non-canonical *FLT3* mutations, which could be classified into three subtypes: non-canonical point mutations (NCPM), insertion/deletion variants causing in-frame amino acid alterations, and other insertions (INS). The prevalence of NCPMs was significantly higher in ALL patients (58.3%) compared to AML patients (11.1%, *p* < 0.001), whereas the incidence of other types of mutations was 16.6% vs. 7.4% (**Figure 1**C–E).

The median length of insertions in AML was 46.5 base pairs (bp) (range: 18–102 bp), significantly greater than the median length in the ALL group, which was 6 bp (range: 3–12 bp, *p* = 0.002). (**Figure 1**F).

### 2.3. Patterns of Co-Occurrence in FLT3 Gene Mutations

**Figure 2:**
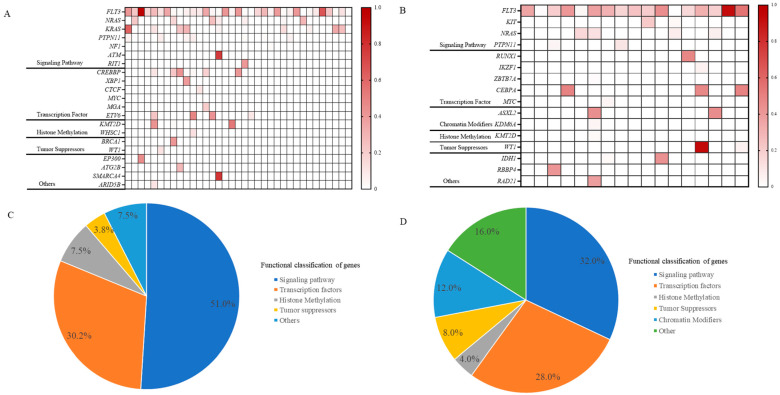
Co-mutation landscape of *FLT3* mutations in ALL (**A**) and in AML (**B**). Proportional distribution of co-mutations in ALL with *FLT3* mutations (**C**) and in AML with *FLT3* mutations (**D**). In Figures (**A**,**B**), the intensity of the color indicates the mutation frequency, with darker colors indicating a higher mutation frequency and lighter colors indicating a lower mutation frequency. Abbreviations: ALL, acute lymphoblastic leukemia; AML, acute myeloid leukemia.

In our investigation of 35 ALL patients with *FLT3* gene mutations, we observed that co-mutations associated with diseases primarily affected signaling pathways and transcription factors, accounting for 51.0% and 30.2% of cases, respectively (**Figure 2**A,C). Specifically, mutations in signaling pathways were identified in *NRAS* (7 patients), *KRAS* (10 patients), and *PTPN11* (7 patients). Transcription factor mutations involved *CREBBP*, *XBP1*, *CTCF*, *MYC*, *MGA*, and *ETV6*. Additionally, mutations in histone methylation and tumor suppressor genes were found in 7.5% and 3.8% of cases, respectively, with sporadic mutations in *EP300*, *ATG2B*, *SMARCA4*, and *ARID5B*. 

Similarly, our findings indicated that signaling pathways and transcription factors each accounted for 32.0% and 28.0% of AML cases with co-mutations, respectively. The principal signaling pathway mutations include *NRAS*, *PTPN11*, and *KIT*, while the transcription factors involved were *RUNX1*, *IKZF1*, *ZBTB7A*, *CEBPA*, and *MYC*. These *FLT3* mutations are also accompanied by histone methylation alterations, observed in 4.0% of cases. A distinctive feature of AML was the prevalent occurrence of chromatin modifiers (12.0%), particularly in genes like *ASXL2* and *KDM6A*. Furthermore, occasional mutations were identified in genes such as *IDH1*, *RBBP4*, and *RAD21* (**Figure 2**B,D). 

### 2.4. FLT3 and Their Related Gene Expressions

**Figure 3:**
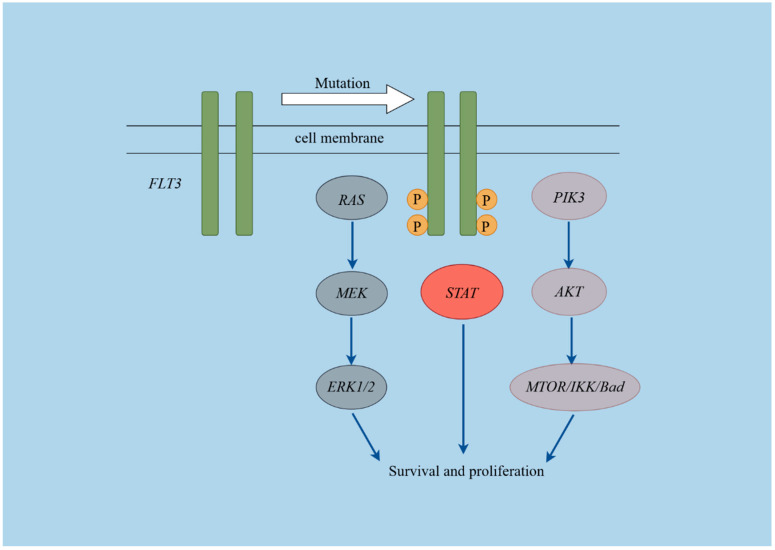
Schematic diagram of the *FLT3* pathways. This figure was created using Figdraw (https://www.figdraw.com/#/, Access Date: 1 September 2024).

Overexpression of *FLT3* may lead to its activation as a receptor and serve as a prognostic marker in pediatric acute leukemia. We analyzed *FLT3* levels and related pathway genes in 206 ALL and 57 AML patients using RNA sequencing data. The expression levels of genes involved in the *FLT3* signaling pathway (**Figure 3**), including *FLT3*, *KRAS*, *HRAS*, *NRAS*, *PIK3CA*, *PIK3CB*, *PIK3CD*, *PIK3R3*, *AKT1*, *AKT2*, *AKT3*, *MTOR*, *CHUK*, *IKBKB*, *IKBKG*, *BAD*, *BRAF*, *RAF1*, *MAP2K1*, *MAP2K2*, *MAPK1*, *MAPK3*, *STAT3*, *STAT5A*, and *STAT5B* were compared. Gene expression levels were measured using fragments per kilobase of exon model per million mapped fragments (FPKM).

In the ALL cohort, the median expression of *FLT3* in the mutant group was 108.02 [85.11, 142.06] FPKM, significantly higher than in the non-mutant group (*p* < 0.001), which had a median expression of 23.11 [9.16, 59.14] FPKM. Concurrently, the expression levels of *HRAS* (2.35 [1.57, 3.16] FPKM vs. 2.77 [2.08, 3.88] FPKM, *p* = 0.044), *PIK3R3* (1.33 [0.69, 1.77] vs. 2.02 [1.03, 3.84] FPKM, *p* = 0.002), *BAD* (3.39 [2.25, 4.52] FPKM vs. 4.28 [3.11, 5.34] FPKM, *p* = 0.018), *MAP2K2* (6.84 [4.03, 9.53] FPKM vs. 8.06 [6.88, 10.10] FPKM, *p* = 0.017), *MAPK3* (5.21 [3.92, 6.62] FPKM vs. 6.01 [5.01, 7.16] FPKM, *p* = 0.028), and *STAT5A* (12.31 [9.28, 15.57] FPKM vs. 14.58 [11.02, 19.61] FPKM, *p* = 0.029) were significantly lower in the mutant group compared to the non-mutant group. No notable differences were observed in the expression levels of the remaining genes between the two groups (Table 1).

Interestingly, significant increases in expression levels were observed in the *FLT3* mutant group compared to the wild-type group for several genes in the AML cohort: *FLT3* (74.77 [54.31, 109.46] vs. 34.56 [20.98, 48.28] FPKM, *p* < 0.001), PIK3CB (23.05 [18.81, 25.80] FPKM vs. 17.60 [13.28, 19.85] FPKM, *p* = 0.006), *AKT1* (9.66 [8.38, 11.61] FPKM vs. 7.40 [5.55, 8.88] FPKM, *p* = 0.003), *MTOR* (9.33 [7.75, 11.35] FPKM vs. 7.70 [6.79, 8.65] FPKM, *p* = 0.006), *BRAF* (13.38 [11.63, 15.12] FPKM vs. 11.37 [9.61, 13.19] FPKM, *p* = 0.015) and *MAPK1* (14.84 [13.07, 17.90] FPKM vs. 12.72 [11.02, 14.92] FPKM, *p* = 0.029), while the expression levels of the other genes did not show significant differences between the two groups (Table 2).

### 2.5. Prognostic Impacts of FLT3 Mutations

With a median follow-up time of 29 ± 1.5 months for OS, among the total 20 patients who died in the ALL cohort, one was found to harbor an *FLT3* mutation. Due to the small number of deaths, the median survival time was not reached. There were no statistically significant differences in OS (96.6% vs. 87.8%, *p* = 0.23) and event-free survival (EFS) (93.6% vs. 69.1%, *p* = 0.12) between the mutation and wild-type groups (**Figure S1A,B**).

Among children with AML, 4 out of the 13 patients who died had an *FLT3* mutation. The presence of the *FLT3* mutation was not associated with significantly inferior outcomes compared to the non-mutation subgroup (OS: 71.5% vs. 70.7%, *p* = 0.86; EFS: 48.6% vs. 52.9%, *p* = 0.98), with a median follow-up time of 28.0 ± 5.9 months for OS (**Figure S1C,D**).

## 3. Discussion

*FLT3*-activating mutations interfere with the differentiation and maturation processes of hematopoietic cells [2]. Recent studies have increasingly demonstrated that *FLT3* inhibitors exert a beneficial effect in a subset of patients; however, the specific nature of *FLT3* mutations that may benefit pediatric ALL and AML patients remains to be determined [3,4,15,23,24]. This study involved a comprehensive clinical, genomic, and transcriptomic evaluation of *FLT3* to elucidate its variations and their pathological impacts in pediatric ALL and AML, thereby informing the use of *FLT3* inhibitors. Our findings indicated that, in patients with *FLT3* mutations, point mutations predominated in ALL, whereas ITD mutations were more common in AML. Further transcriptomic analysis revealed high *FLT3* expression in both conditions but with distinct differences in the expression levels of *FLT3*-related pathway genes. These distinctions suggest that *FLT3* activation led to divergent downstream signaling cascades, which may serve as a critical determinant in the choice of *FLT3* inhibitor therapy.

This investigation provided an extensive overview of the clinical characteristics of ALL and AML harboring *FLT3* mutations utilizing NGS. The *FLT3* mutation rates were found to be 14.4% for ALL and 32.3% for AML, indicating an increase compared to previous studies [5,6], demonstrating the superior sensitivity and enhanced detection capabilities of NGS [25,26]. In the context of ALL, no marked leukocytosis was detected, in concordance with previously published reports [3,16]. A significantly increased tendency for *FLT3* mutations was observed with high hyperdiploidy, consistent with other studies [3,13,16]. However, the significantly increased frequency of *FLT3* mutations in B-ALL observed in this study remains inconclusive [3,5]. Further investigation with larger sample sizes is warranted to clarify the proportions of *FLT3* mutations in B-ALL and T-cell ALL. Strikingly, in our cohort, there was a pronounced inverse correlation between *FLT3* mutations and *ETV6-RUNX1*, suggesting a mutually exclusive relationship between *FLT3* mutation and *ETV6-RUNX1* in ALL tumorigenesis, which may be attributed to the propensity of mutated patients to lack the fusion gene [27]. In AML, the *FLT3* mutation was significantly associated with *NUP98* [28] fusions and indicated a higher tumor burden [4]. Preclinical trials have also shown that mice harboring both *FLT3*-ITD and *NUP98* fusion genes exhibited more aggressive leukemia with a shorter latency period [29]. Additionally, a subtle negative association was noted between *FLT3* mutations and the presence of the *AML1-ETO* translocation, typically representing a low-risk feature [30], indicating diverse contributions to leukemogenesis.

The above analysis detailed the diverse mutational forms of *FLT3* in ALL and AML, along with the expression spectra of *FLT3*-related pathway genes, suggesting differential activation of *FLT3*-related downstream signaling pathways. This assertion can be elaborated from three distinct perspectives. Firstly, among 35 pediatric ALL patients with *FLT3* mutations, various forms such as TKD, ITD, INS, and deletion/insertion were identified, with NCPM being predominant, echoing earlier results [3,6]. In contrast, among 20 AML patients with *FLT3* mutations, 18 exhibited the *FLT3*-ITD subtype, characterized by inserted bases ranging from 18 to 102. These observations emphasized the distinct roles that *FLT3* mutations may play in the pathogenesis of ALL and AML. ITD mutations in the JMD disrupted its autoinhibition by elongating the domain, rather than by an increased number of tyrosine residues, showing distinct structural changes resulting from different amino acid alterations [31]. Concurrently, research has demonstrated that *FLT3* point mutations can modify the substrate specificity of protein kinases [32]. Consequently, we inferred that the distinct mutation patterns in ALL and AML may affect various docking sites and act upon different substrates. Secondly, *FLT3*-ITD mutants induce receptor autophosphorylation and promote interleukin-3-independent growth in Ba/F3 cells, strongly activating STAT5 and *MAPK* pathways. In contrast, ligand-stimulated *FLT3* wild-type activates *AKT* and *MAPK* pathways without affecting *STAT5* [33]. Point mutations in the JMD of *FLT3* in AML exhibited a diminished transforming capability, which was associated with reduced autophosphorylation of the receptor and its downstream target *STAT5* [34]. Chatain et al. elucidated that *FLT3* JMD point mutations and deletions in AML led to phosphorylation of both *STAT3* and *STAT5*, while *FLT3*-ITD mainly activated *STAT5* over *STAT3* [35]. The Y842 mutation in the *FLT3*-ITD background impairs the *RAS*/*ERK* pathway and delays tumor formation [36]. Furthermore, substitutions of tyrosine residues 589 and 591 with phenylalanine in *FLT3*-ITD can disrupt *STAT5* signaling without affecting tyrosine kinase activity, thus preventing the myeloproliferative phenotype in murine bone marrow cells [37]. Collectively, these findings suggest that the precise degree and specific pathways of downstream signaling activation play a crucial role in leukemia development beyond just receptor activation. Thirdly, *FLT3*-related signaling pathways primarily include the *JAK*-*STAT*, *MAPK*, and *PI3K*-*AKT* pathways [2]. We conducted an analysis of the expression levels of related genes. Transcriptome data analysis revealed differential expression profiles of *FLT3*-associated pathway genes in ALL and AML. In ALL, genes in the relevant signaling pathways were downregulated in the mutation group, whereas in AML, they were upregulated, highlighting the differing roles of *FLT3* mutations in the development of these leukemias. This may partially explain why *FLT3*-ITD patients have a poor prognosis and provide robust evidence for the advancement of targeted and precise therapeutic strategies.

The research further revealed that, regardless of whether the cases involved pediatric ALL or AML, *FLT3* mutations did not correspond with a worse prognosis. The underlying reasons in ALL are analyzed as follows. In ALL patients, firstly, it is apparent that patients with *FLT3* mutations frequently present with hyperdiploidy, which is indicative of chemosensitivity and a favorable prognosis [38,39,40]. Secondly, the mutations are primarily point mutations, which generally exerted a less significant influence on downstream signaling pathways due to lower autophosphorylation [34], with only three individuals found to carry the ITD mutation. In AML cases, the prevalence of *FLT3*-ITD mutations markedly increased, reaching up to 90% among patients receiving treatment. The cytological remission rate was 75%, which is similar to the 79.8% total post-induction cytological complete remission rate reported for the Chinese Children’s Leukemia Group (CCLG)-AML-2015 regimen in the Homoharringtonine-based induction group and 75% in the high-risk group [41]. Regarding prognostic outcomes, in the cohort of 18 AML patients harboring *FLT3*-ITD mutations, three individuals withdrew from treatment and were subsequently excluded from the prognostic evaluation. Details of the treatment regimen are illustrated in Table S5. Specifically, three patients terminated their therapy during the induction chemotherapy phase. Of the seven patients who underwent hematopoietic stem cell transplantation (HSCT), it was the incorporation of *FLT3* inhibitors that enabled three of them to achieve complete remission and qualify for HSCT. Ultimately, five patients enjoyed favorable prognoses [9,42], although two died due to transplant-related complications. Conversely, three patients received intensified chemotherapy without *FLT3* inhibitors. This resulted in two deaths due to severe post-chemotherapy infections, and one patient remained in non-remission, thereby missing the opportunity for HSCT, leading to a poor outcome. An additional patient, who received intensive chemotherapy augmented with *FLT3* inhibitors, remained in sustained remission. This aligns closely with recent studies indicating the significant potential of *FLT3* inhibitors for AML patients with *FLT3*-ITD mutations [21,23]. Encouragingly, a report indicated that, following HSCT relapse and the detection of a *FLT3*-TKD mutation, an early T-cell precursor ALL patient achieved a second complete remission and MRD negativity with gilteritinib treatment [43]. Moreover, venetoclax works synergistically with gilteritinib in *FLT3* wild-type high-risk AML by suppressing *MCL-1* [44]. These observations imply that *FLT3* inhibitors may have potential applications in non-*FLT3*-ITD cases. Based on the differential downstream gene expression profiles in our transcriptomic data for ALL and AML, we infer that these benefits from *FLT3* inhibitors may be attributed to more comprehensive mechanisms involving the activation of downstream signaling pathways.

The present study investigated the patterns of concurrent *FLT3* mutations in ALL and AML to identify specific gene–gene interactions that play a key role in the emergence and progression of these diseases. Research into AML has demonstrated that *FLT3* wild-type high-risk patients with mutations in *NPM1*, *DNMT3A*, co-occurring *NPM1*/*DNMT3A*, “activated signaling,” and “DNA methylation” genes showed improved OS with sorafenib maintenance [45]. Additionally, midostaurin has been shown to potentially improve OS and disease-free survival in *FLT3*-ITD AML patients with chromatin-spliceosome mutations [46], and relapsed/refractory *FLT3*-mutated AML patients with *DNMT3A*/*NPM1* co-mutations experienced the most positive outcomes when treated with gilteritinib [47]. These findings imply that genetic patterns are crucial in the pathogenesis and treatment of AML. In our study, the distribution of the top three co-mutated genes in ALL, which were related to signaling pathways, transcription factors, and histone methylation, was similar to the mutational spectrum observed in 219 pediatric ALL [27]. This pattern seemingly suggested that *FLT3* mutations did not have unique co-mutated partner genes. In AML, we found that concomitant mutations exhibited a wide variety of irregular patterns. Due to the limited sample size, it is currently not feasible to investigate the prognostic relevance of *FLT3* mutations accompanied by other genetic mutations through subgroup analysis. It is important to interpret these findings with caution due to the finite sample size. Further data collection is necessary to validate these incidences and to explore the relationships between these genetic factors and prognosis.

Our study has some limitations. Due to its retrospective nature, we did not investigate downstream signaling molecule phosphorylation levels, which are crucial for elucidating the underlying pathogenic mechanisms. Moreover, the single-center design of the study led to limited sample sizes, preventing robust analysis of some genetic pattern subgroups and their relationship with prognosis. Future studies should leverage multi-center data to focus on co-mutations and related signaling pathways. Ultimately, assessing drugs that target these pathways and conducting translational research are expected to enhance treatment outcomes for patients with *FLT3* mutations and other genetic alterations.

## 4. Methods and Materials

### 4.1. Participants and Study Design

A total of 274 de novo ALL and 74 newly diagnosed AML patients were identified at our hospital between January 2019 and January 2023. After excluding those who did not complete comprehensive genetic testing for hematologic tumors, 243 ALL and 62 AML patients were included in the study. Refer to **Figure S2** for a detailed description of the study process. All patients were diagnosed with ALL or AML based on morphology, flow cytometry, immunohistochemistry, and genetic testing, and received either the Chinese Children’s Cancer Group (CCCG)-ALL-2015/2020 protocol or the CCLG-AML-2015/2019 regimen. Detailed treatment protocols are provided in Tables S6 and S7. This study was approved by the Ethics Committee of the Union Hospital of Tongji Medical College, Huazhong University of Science and Technology (No. 2024–0034).

### 4.2. Chemotherapy Protocol

The therapeutic approach for ALL patients was aligned with similar protocols, specifically CCCG-ALL-2015 or CCCG-ALL-2020, with early-stage patients adhering to the 2015 plan. The specific treatment protocols are detailed in Supplemental Tables S6 and S7. Similarly, for AML, chemotherapy regimens are provided in Tables S8 and S9. Information about HSCT treatment for the patients can be found in Table S10.

### 4.3. Comprehensive Genetic Testing for Hematologic Tumors

Genomic DNA was extracted using the QIAamp DNA Blood Mini Kit (TIANGEN Biotech (Beijing) Company Limited, Beijing, China) according to the manufacturer’s instructions. The DNA sample was quantified by Qubit dsDNA BR Assay kit (Nanjing Vazyme Biotech Company Limited, Nanjing, China), and DNA integrity was assessed by agarose gel electrophoresis (Shanghai GeneRay Biotech Company Limited, Shanghai, China). DNA was sheared on the Covaris M220 focused ultrasonicator (Gene Technology (Shanghai) Company Limited, Shanghai, China). All libraries were prepared using the KAPA HTP Library Preparation Kit (Shanghai GeneRay Biotech Company Limited, Shanghai, China) according to the manufacture’s instruction. Fragmented DNA was repaired, 3’dA-tailed, ligated with Illumina adapters, size selected, amplified, and assessed using the Agilent 2100 Bioanalyzer (Agilent Bio (Hangzhou) Company Limited, Hangzhou, Zhejiang Province, China). For a targeted capture library of structural variant and single nucleotide variant analysis, a customized panel of biotinylated oligoprobes (Shanghai GeneRay Biotech Company Limited, Shanghai, China) was designed to capture the likely break regions of genes and hotspot mutational regions of hematological malignancies that were identified in earlier leukemia sequencing studies. The captured DNA library was finally amplified and sequenced on Illumina Novaseq 6000 sequencer (Illumina (China) Scientific Equipment Company Limited. Shanghai, China) for paired reads at 150 bp. The raw data were converted from BCL files to FASTQ format using Illumina CASAVA 1.8 (Illumina (China) Scientific Equipment Company Limited. Shanghai, China). The reads were aligned to the GRCh37/hg19 human genome reference using BWA (Broad Institute of MIT and Harvard, Cambridge, MA, USA), and further processed with samtools, picard, and GATK (Broad Institute, Cambridge, MA, USA) to eliminate duplicate sequences and detect genetic variants. All discovered variants were assessed by consulting databases such as NCBIdbSNP (https://www.ncbi.nlm.nih.gov/snp/ Access Date: January 2019 to January 2023), OMIM (https://www.ncbi.nlm.nih.gov/omim/ Access Date: January 2019 to January 2023), HGMD (http://www.hgmd.org Access Date: January 2019 to January 2023), and NCBI ClinVar (https://www.ncbi.nlm.nih.gov/clinvar/ Access Date: January 2019 to January 2023).

### 4.4. RNA Sequencing

RNA Extraction and Quality Assessment: Bone marrow or blood samples were collected using the PAXgene Blood RNA Tube (Chongqing Yes Service Biomedical Tech, Inc. Chongqing, China). Total RNA was extracted using the MagMAX (Invitrogen Trading (Shanghai) Company Limited. Shanghai, China) for Stabilized Blood Tubes RNA Isolation Kit (Invitrogen Trading (Shanghai) Company Limited. Shanghai, China) (or PAXgene Blood RNA Tubes) (Invitrogen Trading (Shanghai) Company Limited. Shanghai, China). Purification was carried out with the NanodropOne (Thermofisher, Invitrogen Trading (Shanghai) Company Limited. Shanghai, China). RNA integrity was assessed using the Qseq400 system (BIOPTIC, Bioptic Inc Technology Company Limited. New Taipei City, Taiwan, China) with the R1 cartridge, and samples with an RQN of ≥5 were deemed suitable for subsequent library preparation.

Library Preparation and Sequencing: Libraries were prepared using the KAPA RNA HyperPrep Kit (Kapabiosystems, cat.KK8540, Shanghai EAST STAR Science & Technology Import & Export Company Limited, Shanghai, China). rRNA was removed using the KAPA RiboErase (HMR) Kit (Kapabiosystems, cat.KK8482, Shanghai EAST STAR Science & Technology Import & Export Company Limited, Shanghai, China). Library construction was performed with 1 µg of total RNA per sample, following the kit manual’s instructions. Post-construction, library concentration was measured with the Qubit 3.0 Fluorometer (Shanghai Jiahe Biological Technology Company Limited, Shanghai, China.) using the Qubit dsDNA HS Assay Kit (Thermofisher, Invitrogen Trading (Shanghai) Company Limited. Shanghai, China), and library size was assessed using the Qseq400 system (Bioptic Technology Company Limited. New Taipei City, Taiwan, China) with the S2 cartridge. Finally, the libraries were sequenced on the Illumina NovaSeq X Plus platform (Illumina (China) Scientific Instruments Company Limited. Shanghai, China), producing paired-end reads of 150 bp, resulting in over 15 GB of raw data.

### 4.5. Treatment Response

Morphological and immunological evaluation of bone marrow smears was performed on Day 19 and Day 46 for ALL patients, and a bone marrow assessment was conducted on Day 28 of each chemotherapy phase for AML patients. Patients were classified based on their blast cell counts into three categories: M1 (blast cells < 5%), M2 (≥5%, <25%), and M3 (≥25%). Complete remission was defined as bone marrow containing less than 5% blasts accompanied by the regeneration of normal hematopoietic cells and MRD less than 10^−4^.

### 4.6. Follow-Up

All cases were followed up through outpatient visits or telephone consultations, with follow-ups concluding in December 2023. Treatment outcomes were assessed using OS and EFS metrics. OS was defined as the duration from the date of diagnosis to either death or the last follow-up for surviving patients. Relapse, death and loss to follow-up in three situations—due to the progression of the primary disease, definitive abandonment of treatment, or unstable vital signs at the time of departure—were defined as events. However, those lost to follow-up after completing most of their chemotherapy and achieving remission were not defined as events. EFS was defined as the interval from the start of treatment to the occurrence of any event, or to the last follow-up, whichever occurred first.

### 4.7. Statistical Analysis

The baseline characteristics of the patients were presented as frequencies and percentages for categorical data and as medians with interquartile ranges for continuous variables. Categorical variables were analyzed using chi-squared tests or continuity-corrected chi-squared tests, as appropriate. Non-parametric tests were used for continuous variables. OS and EFS were calculated using the Kaplan–Meier method, and comparisons between cohorts were made using the log-rank test. All p-values were calculated using two-tailed tests. A p-value of less than 0.05 was considered statistically significant. Statistical analyses were performed using SPSS (IBM, version 24.0), Prism (version 9), and R (version 4.3.3).

## 5. Conclusions

In conclusion, our study demonstrated that the *FLT3* mutation in ALL did not affect prognosis, unlike in AML, where prognosis could be improved through HSCT and *FLT3* inhibitor therapy. *FLT3* inhibitors might be more therapeutically beneficial for AML compared to ALL when *FLT3* mutations are present, as indicated by the expression profiles of *FLT3*-related pathways.

## Figures and Tables

**Table 1:** Expression levels of *FLT3* and associated pathway genes in ALL.

The Expression Level of Genes (FPKM)	Total (N = 206)	*FLT3* Wild-Type (N = 176)	*FLT3* Mutant Group (N = 30)	*p*
*FLT3* (median [IQR])	31.34 [10.69, 80.33]	23.11 [9.16, 59.14]	108.02 [85.11, 142.06]	<0.001
*KRAS* (median [IQR])	22.18 [16.46, 30.70]	22.05 [16.50, 29.30]	25.03 [16.51, 34.23]	0.476
*HRAS* (median [IQR])	2.72 [2.05, 3.67]	2.77 [2.08, 3.88]	2.35 [1.57, 3.16]	0.044
*NRAS* (median [IQR])	33.62 [27.64, 40.39]	33.46 [28.03, 40.29]	35.90 [23.78, 42.71]	0.865
*PIK3CA* (median [IQR])	19.99 [15.59, 26.74]	20.08 [15.73, 27.08]	18.62 [15.03, 25.17]	0.355
*PIK3CB* (median [IQR])	5.76 [4.50, 7.15]	5.74 [4.49, 7.16]	5.83 [4.53, 7.04]	0.786
*PIK3CD* (median [IQR])	29.48 [20.69, 37.98]	29.63 [21.19, 38.09]	28.03 [18.95, 34.27]	0.409
*PIK3R3* (median [IQR])	1.89 [0.90, 3.49]	2.02 [1.03, 3.84]	1.33 [0.69, 1.77]	0.002
*AKT1* (median [IQR])	7.22 [6.04, 9.22]	7.40 [6.10, 9.23]	7.09 [5.56, 8.63]	0.527
*AKT2* (median [IQR])	9.83 [8.21, 11.75]	10.18 [8.33, 11.79]	8.53 [7.36, 10.89]	0.058
*AKT3* (median [IQR])	7.01 [2.77, 12.78]	6.91 [2.85, 12.67]	8.50 [2.71, 15.05]	0.510
*MTOR* (median [IQR])	9.90 [8.38, 11.71]	9.98 [8.53, 11.76]	9.66 [7.94, 10.84]	0.267
*CHUK* (median [IQR])	8.83 [7.23, 10.21]	8.78 [7.22, 10.19]	9.35 [8.38, 10.58]	0.256
*IKBKB* (median [IQR])	7.83 [6.60, 9.50]	7.87 [6.64, 9.61]	7.66 [6.39, 8.65]	0.442
*IKBKG* (median [IQR])	0.65 [0.50, 0.97]	0.64 [0.50, 0.90]	0.79 [0.51, 1.19]	0.105
*BAD* (median [IQR])	4.20 [3.04, 5.28]	4.28 [3.11, 5.34]	3.39 [2.25, 4.52]	0.018
*BRAF* (median [IQR])	15.13 [12.18, 18.94]	15.15 [12.25, 18.98]	13.68 [11.77, 18.74]	0.571
*RAF1* (median [IQR])	19.67 [16.29, 24.23]	20.09 [16.39, 24.41]	18.88 [14.94, 21.54]	0.108
*MAP2K1* (median [IQR])	11.16 [8.73, 14.23]	11.08 [8.70, 13.32]	11.90 [8.77, 16.70]	0.323
*MAP2K2* (median [IQR])	8.00 [6.54, 9.93]	8.06 [6.88, 10.10]	6.84 [4.03, 9.53]	0.017
*MAPK1* (median [IQR])	15.53 [13.36, 18.54]	15.53 [13.42, 18.67]	15.54 [13.16, 17.06]	0.378
*MAPK3* (median [IQR])	5.93 [4.88, 7.14]	6.01 [5.01, 7.16]	5.21 [3.92, 6.62]	0.028
*STAT3* (median [IQR])	13.76 [11.41, 19.61]	13.76 [11.21, 19.13]	14.15 [12.30, 20.44]	0.495
*STAT5A* (median [IQR])	14.12 [10.23, 19.31]	14.58 [11.02, 19.61]	12.31 [9.28, 15.57]	0.029
*STAT5B* (median [IQR])	16.90 [12.38, 21.67]	17.59 [12.63, 22.01]	14.92 [11.93, 18.83]	0.069

**Table 2:** Expression levels of *FLT3* and associated pathway genes in AML.

The Expression Level of Genes (FPKM)	Total (N = 57)	*FLT3* Wild-Type (N = 38)	*FLT3* Mutant Group (N = 19)	*p*
*FLT3* (median [IQR])	44.22 [26.32, 68.30]	34.56 [20.98, 48.28]	74.77 [54.31, 109.46]	<0.001
*KRAS* (median [IQR])	16.35 [13.13, 19.84]	16.53 [13.46, 19.49]	15.84 [12.70, 22.22]	0.852
*HRAS* (median [IQR])	2.96 [2.43, 3.89]	2.77 [2.11, 3.77]	3.17 [2.82, 3.91]	0.123
*NRAS* (median [IQR])	29.83 [24.84, 33.57]	29.86 [25.28, 33.55]	28.48 [23.02, 33.50]	0.813
*PIK3CA* (median [IQR])	10.25 [8.22, 12.43]	10.46 [9.22, 13.13]	9.06 [7.32, 11.24]	0.09
*PIK3CB* (median [IQR])	18.85 [14.46, 23.19]	17.60 [13.28, 19.85]	23.05 [18.81, 25.80]	0.006
*PIK3CD* (median [IQR])	18.70 [15.47, 24.77]	18.50 [15.39, 24.73]	19.53 [16.52, 25.39]	0.813
*PIK3R3* (median [IQR])	0.26 [0.18, 0.57]	0.26 [0.18, 0.66]	0.26 [0.16, 0.44]	0.542
*AKT1* (median [IQR])	8.32 [6.19, 10.41]	7.40 [5.55, 8.88]	9.66 [8.38, 11.61]	0.003
*AKT2* (median [IQR])	7.31 [5.61, 8.81]	7.32 [5.57, 8.72]	7.28 [6.35, 8.82]	0.531
*AKT3* (median [IQR])	1.66 [1.00, 4.42]	1.52 [1.01, 3.34]	2.50 [1.00, 5.00]	0.379
*MTOR* (median [IQR])	8.02 [7.21, 9.56]	7.70 [6.79, 8.65]	9.33 [7.75, 11.35]	0.006
*CHUK* (median [IQR])	7.63 [6.33, 9.19]	7.62 [6.29, 8.80]	7.82 [6.36, 9.82]	0.542
*IKBKB* (median [IQR])	7.96 [6.63, 9.41]	7.59 [6.39, 9.54]	8.55 [7.81, 9.36]	0.128
*IKBKG* (median [IQR])	0.72 [0.56, 0.88]	0.71 [0.56, 0.87]	0.76 [0.61, 0.91]	0.488
*BAD* (median [IQR])	3.47 [2.58, 4.20]	3.12 [2.44, 4.05]	3.76 [2.90, 4.61]	0.108
*BRAF* (median [IQR])	12.03 [10.37, 14.27]	11.37 [9.61, 13.19]	13.38 [11.63, 15.12]	0.015
*RAF1* (median [IQR])	18.03 [15.47, 20.86]	17.99 [15.39, 20.16]	18.44 [17.02, 21.18]	0.623
*MAP2K1* (median [IQR])	10.33 [7.56, 13.11]	11.78 [7.00, 14.80]	9.87 [8.22, 11.14]	0.335
*MAP2K2* (median [IQR])	9.76 [7.95, 11.72]	9.30 [7.55, 11.59]	10.04 [9.58, 11.68]	0.123
*MAPK1* (median [IQR])	13.25 [11.09, 16.73]	12.72 [11.02, 14.92]	14.84 [13.07, 17.90]	0.029
*MAPK3* (median [IQR])	6.65 [5.51, 7.69]	6.95 [5.50, 7.99]	6.19 [5.81, 7.25]	0.416
*STAT3* (median [IQR])	26.38 [22.24, 30.81]	26.59 [22.31, 30.70]	25.97 [22.98, 30.17]	0.879
*STAT5A* (median [IQR])	28.60 [22.79, 34.60]	29.87 [21.83, 35.70]	26.04 [23.79, 30.46]	0.509
*STAT5B* (median [IQR])	20.34 [17.39, 23.76]	20.43 [17.92, 23.75]	20.34 [17.29, 23.88]	0.787

Abbreviations: AML, acute myeloid leukemia; IQR, interquartile range. Non-parametric test is used for statistical analysis.

## Data Availability

Requests for research data can be made through correspondence upon reasonable request.

## Supplementary Materials

The following supporting information can be downloaded at: https://www.mdpi.com/article/10.3390/ijms25179581/s1.
