# Supplementary material for: Distinct FLT3 Pathways Gene Expression Profiles in Pediatric De Novo Acute Lymphoblastic and Myeloid Leukemia with FLT3 Mutations: Implications for Targeted Therapy
Source: Int J Mol Sci. 2024 Sep 4;25(17):9581. doi: 10.3390/ijms25179581 (PMC11395013; doi:10.3390/ijms25179581)
Supplement: Supplementary file 1 [file ijms-25-09581-s001.zip › Supplement Figures S1 and S2.pdf]

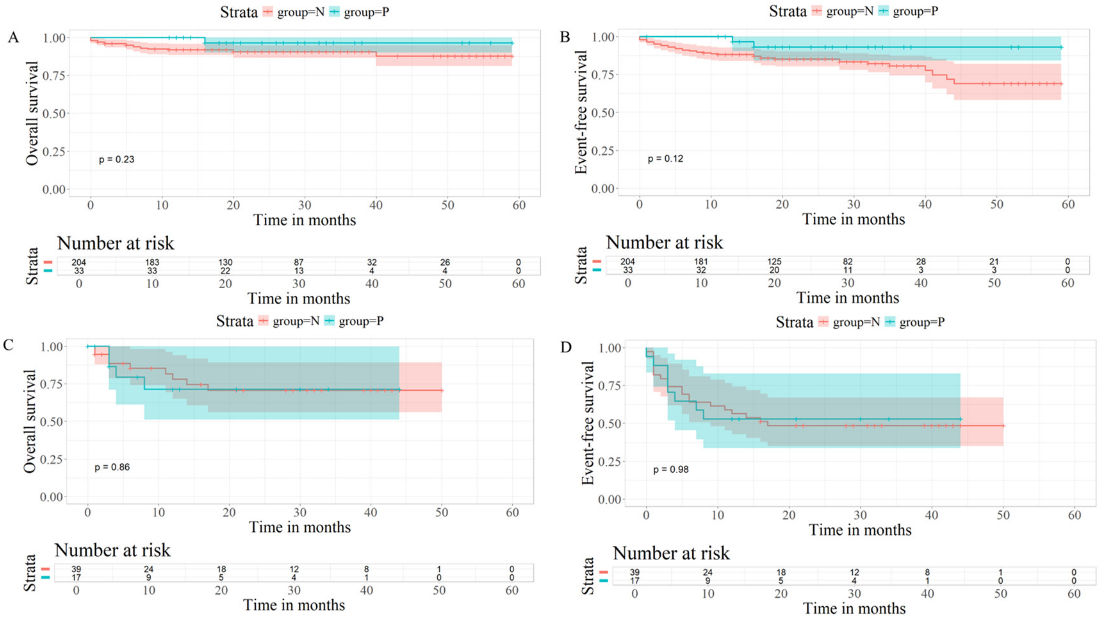

Figure S1 Overall survival of ALL patients with *FLT3* mutant group and wild-type group (A). Event-free survival of ALL patients with *FLT3* mutant group and wild-type group (B). Overall survival of AML patients with *FLT3* mutant group and wild-type group (C). Event-free survival of AML patients with *FLT3* mutant group and wild-type group (D).

Abbreviations: ALL, acute lymphoblastic leukemia; AML, acute myeloid leukemia. “N” represents the *FLT3* wild-type group, while “P” represents the *FLT3* mutation group.

A

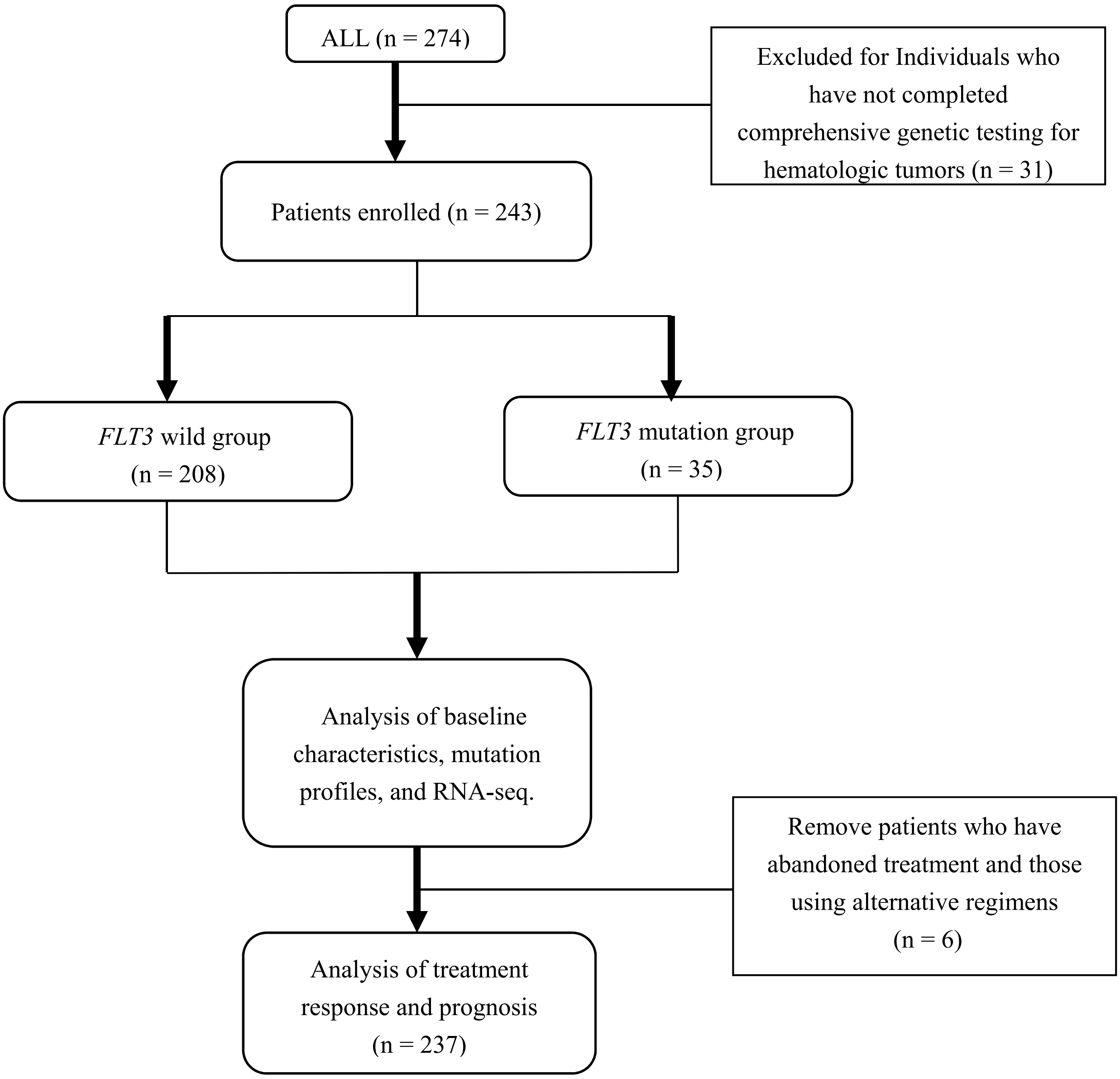
ALL (n = 274)
Excluded for Individuals who have not completed comprehensive genetic testing for hematologic tumors (n = 31)
Patients enrolled (n = 243)
FLT3 wild group (n = 208)
FLT3 mutation group (n = 35)
Analysis of baseline characteristics, mutation profiles, and RNA-seq.
Remove patients who have abandoned treatment and those using alternative regimens (n = 6)
Analysis of treatment response and prognosis (n = 237)

B

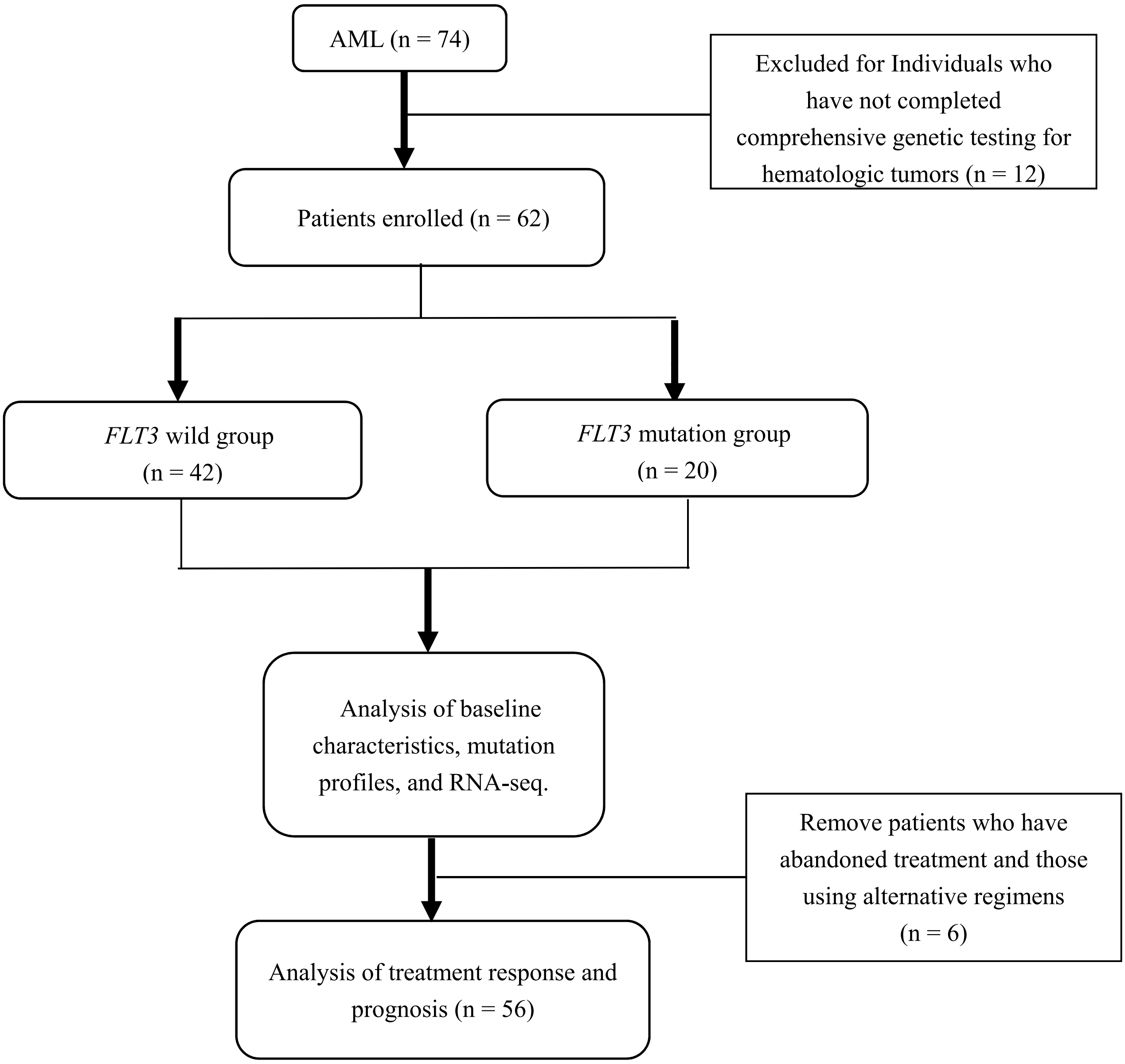

FigureS2 Flowchart of the study process for patients with ALL(A) and AML(B).
Abbreviations: ALL, acute lymphoblastic leukemia; RNA-seq, RNA sequencing.
AML, acute myeloid leukemia.
