# Supplementary material for: Distinct FLT3 Pathways Gene Expression Profiles in Pediatric De Novo Acute Lymphoblastic and Myeloid Leukemia with FLT3 Mutations: Implications for Targeted Therapy
Source: Int J Mol Sci. 2024 Sep 4;25(17):9581. doi: 10.3390/ijms25179581 (PMC11395013; doi:10.3390/ijms25179581)
Supplement: Supplementary file 1 [file ijms-25-09581-s001.zip › Supplement Tables S1-S10.pdf]

Table S1 Pretreatment patient characteristics in ALL.

Characteristics	Total (n = 243)	*FLT3* Wild-type group (n = 208)	*FLT3* Mutant-group (n = 35)	*P*
Gender, n (%)				0.475
Male	152(62.6)	132(63.5)	20 (57.1)	
Female	91(37.4)	76 (36.5)	15(42.9)	
Age(y)(median [IQR])	5.20 [3.50, 9.05]	5.45 [3.48, 9.11]	4.60 [3.80, 6.80]	0.404
Chemotherapy protocol, n (%)				0.587
CCCG-ALL-2015	44(18.6)	39(19.1)	5(15.2)	
CCCG-ALL-2020	193 (81.4)	165(80.9)	28 (84.8)	
Immunophenotyping, n (%)				0.043#
B-lineage	215 (88.5)	180(86.5)	35 (100.0)	
T-lineage	28 (11.5)	28 (13.5)	0 (0.0)	
Genetics (%)				
Hyperdiploid	48(19.8)	31(14.9)	17(48.6)	< 0.001
Hypodiploid	4(1.6)	4(1.9)	0(0)	0.905#
MLL rearrangement	12(4.9)	9(4.3)	3(8.6)	0.515#
ETV6-RUNX1	39(16.0)	39(18.8)	0(0)	0.005
BCR-ABL1	19(7.8)	19(9.1)	0(0)	0.128#
TCF3-PBX1	13(5.3)	13(6.3)	0(0)	0.265#
Blast% (median [IQR]) *	0.88 [0.78, 0.94]	0.88 [0.78, 0.94]	0.92 [0.84, 0.94]	0.163
WBC (median [IQR])	10.38[3.64, 60.44]	11.32 [3.79, 65.50]	5.47[3.12, 18.00]	0.072
Hb (median [IQR])	76.0 [63.0, 91.0]	76.0[65.0, 91.0]	68.5 [53.0, 85.8]	0.133
PLT (median [IQR])	52.0[28.0, 113.0]	51.0[28.0, 106.5]	61.0[33.8, 122.5]	0.434

Abbreviations: ALL, acute lymphoblastic leukemia; CCCG-ALL-2015, Chinese Children's Cancer Group-ALL-2015; CCCG-ALL-2020, Chinese Children's Cancer Group-ALL-2020; IQR, Interquartile range; WBC, white blood cell; Hb, hemoglobin; PLT, platelet.

* Marrow blast.

Non-parametric test is used for continuous variables.

#Continuity-corrected chi-squared tests, the remaining categorical variables were analyzed using the chi-square test.

Table S2 Evaluation of response to chemotherapy in ALL.

Characteristics	Total (n=237)	*FLT3* Wild-type (n=204)	*FLT3* Mutant-group (n=33)	*P*
D19 cytology, n (%)				
M1	210 (89.3)	179 (88.6)	31 (93.9)	0.538#
M2/M3	25 (10.7)	23 (11.4)	2 (6.1)	
D19 MRD, n (%)				
<0.01%	99 (41.9)	92 (45.3)	7 (21.2)	0.009
≥0.01%	137 (58.1)	111 (54.7)	26 (78.8)	
D46 cytology, n (%)				
M1	215 (94.7)	182 (93.8)	33 (100.0)	0.295#
M2/M3	12 (5.3)	12 (6.2)	0 (0.0)	
D46 MRD, n (%)				
<0.01%	207 (90.4)	176 (89.8)	31 (93.9)	0.669#
≥0.01%	22 (9.6)	20 (10.2)	2 (6.1)	

Abbreviations: ALL, acute lymphoblastic leukemia; MRD, minimal residual disease

#Continuity-corrected chi-squared tests, the remaining categorical variables were analyzed using the chi-square test.

Table S3 Pretreatment patient characteristics in AML.

Characteristics	Total (n = 62)	*FLT3* Wild-type (n = 42)	*FLT3* Mutant-group (n = 20)	*P*
Gender, n (%)				0.374
Male	39 (62.9)	28 (66.7)	11 (55.0)	
Female	23 (37.1)	14 (33.3)	9 (45.0)	
Age (y) (median [IQR])	8.00[5.20, 11.00]	8.05[4.58, 10.75]	8.00 [5.80, 11.00]	0.662
Chemotherapy protocol, n (%)				0.432#
CCLG-AML-2015	4(7.1)	4(10.0)	0(0)	
CCLG-AML-2019	52(92.9)	36(90.0)	17(100)	
Immunophenotyping, n (%)				0.143
Unspecified	12 (19.4)	7 (16.7)	5 (25.0)	
M1	3 (4.8)	1 (2.4)	2 (10.0)	
M2	24 (38.7)	20 (47.6)	4 (20.0)	
M4	10 (16.1)	7 (16.7)	3 (15.0)	
M5	11 (17.7)	5 (11.9)	6 (30.0)	
M7	2 (3.2)	2 (4.8)	0 (0.0)	
Genetics (%)				
AML1-ETO	20(32.3)	18(42.9)	2(10.0)	0.010
CBFB-MYH11	8(12.9)	6(14.3)	2(10.0)	0.948#
MLL rearrangement	6(9.7)	5(11.9)	1(5.0)	0.689#
NUP98 rearrangement	8(12.9)	2(4.8)	6(30.0)	0.018#
t (8;21)	13(21.0)	11(26.2)	2(10.0)	0.258#
Inv (16)	5(8.1)	3(7.1)	2(10)	1.000#
complex karyotype	5(8.1)	4(9.5)	1(5)	0.846#
Blast (median [IQR]) *	0.66 [0.46, 0.80]	0.57 [0.44, 0.77]	0.79 [0.52, 0.84]	0.076
WBC (median [IQR])	42.20[11.99, 109.00]	20.36 [8.90, 55.39]	82.90 [47.05, 189.76]	0.001
Hb (median [IQR])	79.00 [60.00, 92.00]	79.50 [61.50, 89.00]	73.00 [59.00, 98.00]	0.732
PLT (median [IQR])	40.00 [20.00, 79.00]	34.50 [18.00, 71.00]	52.00 [30.00, 99.00]	0.125

Abbreviations: AML, acute myeloid leukemia; CCLG-ALL-2015, Chinese Children's Leukemia Group-AML-2015; CCLG-ALL-2019, Chinese Children's Leukemia Group-AML-2019; IQR, interquartile range; WBC, white blood cell; Hb, hemoglobin; PLT, platelet.

* Marrow blast.

Non-parametric test is used for continuous variables.

#Continuity-corrected chi-squared tests, the remaining categorical variables were analyzed using the chi-square test.

Table S4 Evaluation of response to chemotherapy in AML

Characteristics	Total (n=56)	*FLT3* Wild-type (n=39)	*FLT3* Mutant-group (n=17)	*P*
The first D28 cytology, n (%)				0.117#
M1	32 (65.3)	24 (72.8)	8 (50.0)	
M2/M3	17 (34.7)	9 (27.2)	8 (50.0)	
The first D28 MRD, n (%)				0.282
<0.01%	22 (42.3)	17 (47.2)	5 (31.3)	
≥0.01%	30 (57.7)	19 (52.8)	11 (68.8)	
The second D 28 cytology, n (%)				0.852#
M1	34 (81.0)	25 (83.3)	9 (75.0)	
M2/M3	8(19.0)	5 (16.7)	3 (25.0)	
The second D28 MRD, n (%)				0.562#
<0.01%	30 (71.4)	22 (75.9)	8 (61.5)	
≥0.01%	12 (28.6)	7 (24.1)	5 (38.5)	

Abbreviations: AML, acute myeloid leukemia; MRD, minimal residual disease.

#Continuity-corrected chi-squared tests, the remaining categorical variables were analyzed using the chi-square test.

Table S5 Treatment regimens for pediatric AML patients with ITD mutations.

Patient ID	chemotherapy regimens	Outcome	Causes of death
NO.1	DAH+IAH+FLAG	died	complications related to a severe infection
NO.2	DAH+IAH+IA	died	complications related to a severe infection
NO.8	DAH+IAH+HSCT with oral sorafenib and venclexta	died	Transplant-related complications
NO.3	DAH+IAH+HSCT	Survive	/
NO.4	DAH+IAH+HSCT	Survive	/
NO.5	DAH+IAH	Survive	/
NO.6	DAH+IAH+IA+HSCT with oral sorafenib	Survive	/
NO.7	DAH+IAH++FLAG+HSCT	died	Transplant-related complications
NO.9	DAH+VAP with Gilteritinib +HSCT	Survive	/
NO.10	DAH+FLAG+HSCT	Survive	/
NO.14	DAH+IAH+CLAG+CLAG and venclexta+others	Give up	Persisting without achieving complete remission
NO.11	DAH+IAH+decitabine+ sorafenib+ venclexta	Survive	/
NO.12	DAH+IAH	Give up	/
NO.13	DAH+IAH+ decitabine+ sorafenib+ venclexta	Give up	/
NO.15	DAH	Give up	/

Abbreviations: AML, acute myeloid leukemia; ITD, internal tandem duplication;

DAH: DNR, daunorubicin; Ara-c, cytarabine; HHT, homoharringtonine;

IAH: IDA, idarubicin; Ara-c, cytarabine; HHT, homoharringtonine;

IA: IDA, idarubicin; Ara-c, cytarabine;

HSCT: hematopoietic stem cell transplantation;

FLAG: Flu, fludarabine; Ara-c, cytarabine; G-CSF, granulocyte colony-stimulating factor;

VAP: V, venclexta; Ara-c, cytarabine; P, palbociclib;

CLAG: CL, cladribine, Ara-c, cytarabine, G-CSF, granulocyte colony-stimulating factor;

Others: treated at other hospitals, specific treatment regimen unknown.

Table S6 CCCG-ALL-2015 protocol treatment plan framework for different risk stratifications.

Risk stratification	Induction remission			Consolidation	Continuation therapy				Maintenance chemotherapy 1	Maintenance chemotherapy 2
	PVDL	CAT	CAT+		interim maintenance 1	Reinduction 1	interim maintenance 2	Reinduction 2		
SR	Pred<br>VCR<br>DNR<br>PEG-ASP<br>IT	CTX<br>Ara-c<br>6-MP<br>IT	CTX<br>Ara-c<br>6-MP<br>VCR<br>PEG-ASP<br>IT	HD-MTX<br>6-MP<br>IT	6-MP<br>Dex<br>VCR<br>IT<br>MTX	Dex<br>VCR<br>DNR<br>L-ASP	6-MP<br>MTX<br>Dex<br>VCR<br>IT	Dex<br>VCR<br>DNR<br>L-ASP	MTX<br>6-MP<br>Dex<br>VCR<br>IT	MTX<br>6-MP<br>Dex*<br>VCR*

Risk stratification	Induction remission			Consolidation	Continuation therapy		Maintenance chemotherapy 1	Maintenance chemotherapy 2
	PVDL	CAT	CAT+		interim maintenance	Reinduction		
IR/HR	Pred<br>VCR<br>DNR<br>PEG-ASP<br>IT	CTX<br>Ara-c<br>6-MP<br>IT	CTX<br>Ara-c<br>6-MP<br>VCR<br>PEG-ASP<br>IT	HD-MTX<br>6-MP<br>IT	Dex<br>DNR<br>VCR<br>6-MP<br>PEG-ASP<br>IT	Dex<br>VCR<br>HD-Ara-c<br>PEG-ASP<br>IT	MTX<br>6-MP<br>CTX<br>VCR<br>Ara-c<br>Dex<br>IT	MTX<br>6-MP<br>CTX<br>Ara-c<br>Dex*<br>VCR*

Abbreviations: ALL, acute lymphoblastic leukemia; Pred, prednisone; VCR, vincristine; DNR, daunorubicin; PEG-ASP, pegaspargase; IT, intrathecal chemotherapy; CTX, cyclophosphamide; Ara-c, cytarabine; 6-MP, 6-mercaptopurine; Dex, dexamethasone; HD-MTX, high-dose methotrexate; L-ASP, l-asparaginase; SR, standard risk; IR, intermediate risk; HR, high risk; * Group A.

Note: CCCG-ALL-2015 was Chinese Children's Cancer Group acute lymphoblastic leukaemia 2015 protocol: CAT+ for T-cell acute lymphoblastic leukemia or day 19 minimal residual disease ≥ 1%. Group A: dexamethasone and vincristine were added to the last 7 cycles of maintenance therapy; Group B: dexamethasone and vincristine were not added for the last 7 cycles of treatment. The HR group should receive allogeneic transplantation therapy after completion of HD-MTX, otherwise the same as the IR group.

Table S7 CCCG-ALL-2020 protocol treatment plan framework for different risk stratifications.

Risk stratification	Induction remission			Consolidation	Continuation therapy			Maintenance chemotherapy 1	Maintenance chemotherapy 2
					Reinduction 1	interim maintenance	Reinduction 2		
SR	Pred<br>VCR<br>DNR<br>PEG-ASP<br>IT	CTX<br>Ara-c<br>6-MP<br>IT	CTX<br>Ara-c<br>6-MP<br>VCR<br>PEG-ASP<br>IT	HD-MTX<br>6-MP<br>IT	Dex<br>DNR<br>VCR<br>PEG-ASP<br>IT	6-MP<br>MTX<br>VCR<br>Dex<br>6-MP<br>IT	6-MP<br>Dex<br>VCR<br>PEG-ASP<br>IT<br>6-MP	MTX<br>6-MP<br>VCR<br>Dex<br>IT	MTX<br>6-MP

Risk stratification	Induction remission			Consolidation	Continuation therapy		Maintenance chemotherapy 1	Maintenance chemotherapy 2
					interim maintenance	Reinduction		
IR/<br>HR	VCR<br>DNR<br>PEG-ASP<br>Pred*<br>Dex*<br>IT	CTX<br>Ara-c<br>6-MP<br>IT	CTX<br>Ara-c<br>6-MP<br>VCR<br>PEG-ASP<br>IT	HD-MTX<br>6-MP<br>IT	Dex<br>DNR<br>VCR<br>6-MP<br>PEG-ASP<br>IT	Dex<br>VCR<br>HD-Ara-c<br>PEG-ASP<br>IT	MTX<br>6-MP<br>CTX<br>VCR<br>Ara-c<br>Dex<br>IT	MTX<br>6-MP<br>CTX<br>Ara-c

Abbreviations: ALL, acute lymphoblastic leukemia; Pred, prednisone; VCR, vincristine; DNR, daunorubicin; PEG-ASP, pegaspargase; IT, intrathecal chemotherapy; CTX, cyclophosphamide; Ara-c, cytarabine; 6-MP, 6-mercaptopurine; Dex, dexamethasone; HD-MTX, high-dose methotrexate; SR, standard risk; IR, intermediate risk; HR, high risk.

Note: CCCG-ALL-2020 was Chinese Children's Cancer Group acute lymphoblastic leukaemia 2020 protocol. * Patients younger than 10 years old with T-cell acute lymphoblastic leukemia all received dexamethasone, while the remaining patients were randomly assigned to receive either dexamethasone or prednisone chemotherapy. CAT+ for T cell ALL or day 19 minimal residual disease ≥ 0.01%. Patients in the SR group were randomized to receive either 2 or 4 courses of HD-MTX.

Table S8 CCLG-AML-2015 protocol treatment plan framework for different risk stratifications.

Risk stratification	Induction remission		Consolidation				Maintenance chemotherapy	
							A group	B group
SR	Ara-c<br>DNR<br>VP-16*<br>HHT*<br>IT	Ara-c<br>IDA<br>VP-16*<br>HHT*<br>IT	Mit<br>Ara-c<br>IT	HHT<br>Ara-c<br>IT	Ara-c<br>L-ASP<br>IT		Ara-c<br>6-MP<br>IT	ATRA<br>6-MP<br>IT
Risk stratification	Induction remission		Consolidation				Maintenance chemotherapy	
							A group	B group
IR/HR	Ara-c<br>DNR<br>VP-16*<br>HHT<br>IT	Ara-c<br>IDA<br>VP-16*<br>HHT<br>IT	Mit<br>Ara-c<br>IT	HHT<br>Ara-c<br>IT	Ara-c<br>L-ASP<br>IT	HHT#<br>Ara-c#<br>IT#	Ara-c<br>6-MP<br>IT	ATRA<br>6-MP<br>IT

Abbreviations: AML, acute myeloid leukemia; Ara-c, cytarabine; DNR, daunorubicin, VP-16, etoposide; HHT, homoharringtonine; IT, intrathecal chemotherapy; IDA, idarubicin; Mit, mitoxantrone; 6-MP, 6-mercaptopurine; ATRA, all-trans retinoic acid; SR, standard risk; IR, intermediate risk; HR, high risk. *Randomly assign them to either the Etoposide group or the HHT group. # Indicated exclusively for HR patients who are unsuitable candidates for hematopoietic stem cell transplantation.

Note: CCLG-AML-2015 was Chinese Children's Leukemia Group acute myeloid leukemia 2015 protocol. For HR pediatric patients, it is recommended to proceed with hematopoietic stem cell transplantation following the first course of consolidation therapy. During the maintenance phase, patients are randomly assigned into two groups to receive different treatment regimens.

Table S9 CCLG-AML-2019 protocol treatment plan framework for different risk stratifications.

<table>
<tr><td rowspan="2">Risk stratification</td><td colspan="2" rowspan="2">Induction remission</td><td colspan="4" rowspan="2">Consolidation</td><td colspan="2">Maintenance chemotherapy</td></tr>
<tr><td>A group</td><td>B group</td></tr>
<tr><td>SR</td><td>Ara-c
DNR
HHT
IT</td><td>Ara-c
IDA
HHT
IT</td><td>Mit
Ara-c
IT</td><td>HHT
Ara-c
IT</td><td colspan="2">Ara-c
L-ASP
IT</td><td>6-MP
Ara-c
IT</td><td>ATRA
RIF
IT</td></tr>
<tr><td rowspan="2">Risk stratification</td><td colspan="2" rowspan="2">Induction remission</td><td colspan="4" rowspan="2">Consolidation</td><td colspan="2">Maintenance chemotherapy</td></tr>
<tr><td>A group</td><td>B group</td></tr>
<tr><td>IR/HR</td><td>Ara-c
DNR
HHT
IT</td><td>Ara-c
IDA
HHT
IT</td><td>Mit
Ara-c
IT</td><td>HHT
Ara-c
IT</td><td>Ara-c
L-ASP
IT</td><td>HHT#
Ara-c#
IT#</td><td>6-MP
Ara-c
IT</td><td>ATRA
RIF
IT</td></tr>
</table>

Abbreviations: AML, acute myeloid leukemia; Ara-c, cytarabine; DNR, daunorubicin, VP-16, etoposide; HHT, homoharringtonine; IDA, idarubicin; Mit, mitoxantrone; 6-MP, 6-mercaptopurine; ATRA, all-trans retinoic acid; RIF, realgar-indigo naturalis formula. SR, standard risk; IR, intermediate risk; HR, high risk.

Indicated exclusively for high-risk patients who are unsuitable candidates for hematopoietic stem cell transplantation.

Note: CCLG-AML-2019 was Chinese Children's Leukemia Group acute myeloid leukemia 2015 protocol. For high-risk pediatric patients, it is recommended to proceed with hematopoietic stem cell transplantation following the first course of consolidation therapy. During the maintenance phase, patients are randomly assigned into two groups to receive different treatment regimens.

Table S10 Information about HSCT treatment for the patients.

Patient ID	Diagnosis	conditioning regimen	GVHD prophylaxis
N1	AML	DAC+Ara-c+BU+CTX	ATG+MTX+FK506+MMF+anti-CD25 MoAb
N2*	AML	ClA+Ara-c+G-CSF+BU+CTX	ATG+MTX+FK506+MMF+ anti-CD25 MoAb
N3	AML	CLA+Ara-c+BU+CTX+ MeCCNU+FLU	ATG+ CSA+MMF
N4	AML	DAC+Ara-c+BU+ CTX+ MeCCNU+FLU	ATG+CSA+MMF
N5	AML	CLA+Ara-c+G-CSF+BU+CTX	ATG+MTX+FK506+MMF+anti-CD25 MoAb
N7	AML	CLA+Ara-c+BU+CTX+MeCCNU+FLU	ATG+CSA+MMF
N8	AML	CLA+Ara-c+G-CSF +BU +CTX	ATG+MTX+CSA+MMF+anti-CD25 MoAb
N9	AML	CLA+Ara-c+G-CSF+BU+CTX	ATG+MTX+FK506+MMF+anti-CD25 MoAb
N10	AML	CLA+Ara-c+G-CSF+BU+CTX	ATG+MTX+FK506+MMF+anti-CD25 MoAb
N11*	AML	CLA+Ara-c+BU+CTX+MeCCNU	MTX+ FK506+MMF
N12*	AML	DAC+Ara-c+BU+CTX+MeCCNU+FLU	ATG+CSA +MMF
N13	AML	DAC+Ara-c+BU+CTX+MeCCNU+FLU	ATG+CSA+MMF
N14	AML	CLA+Ara-c+CTX+FLU+MeCCNU+BU	ATG+CSA+MMF
N15	AML	CLA+Ara-c+BU+CTX+MeCCNU	MTX+ FK506+MMF+ anti-CD25 MoAb
N16	AML	CLA+Ara-c+BU+CTX+FLU+MeCCNU	ATG+CSA +MMF
N17	AML	CLA+Ara-c+BU+CTX+MeCCNU	ATG+MTX+ FK506 +MMF+ anti-CD25 MoAb
N18	AML	DAC+Ara-c+BU+CTX+MeCCNU	ATG+MTX+CSA+MMF+ anti-CD25 MoAb
N19*	AML	DAC+Ara-c+BU+CTX	ATG +MTX+CSA+MMF + anti-CD25 MoAb
N20*	AML	CLA+Ara-c+CTX +BU +FLU	ATG+CSA+MMF
N21*	AML	CLA+Ara-c+CTX+BU +FLU	ATG+CSA+MMF
N22*	AML	CLA+Ara-c+CTX+BU +FLU	ATG+CSA+MMF
N23	AML	DAC+BU+CTX+MeCCNU+FLU	CSA+MMF
N26	ALL	BU+VP16+CTX	ATG+MTX +FK506+MMF+ anti-CD25 MoAb
N27	ALL	BU+VP16+CTX	ATG+MTX +FK506+MMF+anti-CD25 MoAb
N29	ALL	BU+VP16+CTX	ATG+MTX+FK506+MMF+anti-CD25 MoAb
N31*	ALL	BU+VP16+CTX	ATG+MTX+FK506+MMF+anti-CD25 MoAb

Abbreviation: HSCT, hematopoietic stem cell transplantation; ALL, acute lymphoblastic leukemia; AML, acute myeloid leukemia; GVHD, graft-versus-host disease; DAC, decitabine; Ara-c, cytarabine; BU, busulfan; CLA, cladribine; CTX, cyclophosphamide; FLU, fludarabine; MeCCNU, semustine; ATG, anti-Thymocyte globulin; MTX, methotrexate; FK506, tacrolimus; MMF,

mycophenolate mofetil; anti-CD25 moAb, anti-CD25 monoclonal antibody; CSA, ciclosporin A; VP16, etoposide;
* patient with *FLT3* mutations.
Note: N6, N24, N25, N28, N30 represents transplantation at another hospital; specific information is not available.
